# Remdesivir-induced symmetrical drug–related intertriginous and flexural exanthema (SDRIFE)? A case report with review of the literature

**DOI:** 10.1007/s00228-020-02955-4

**Published:** 2020-08-06

**Authors:** Johannes Heck, Dirk O. Stichtenoth, Roland Mettin, Jörg Jöckel, Christoph Bickel, Benjamin Krichevsky

**Affiliations:** 1 Institute for Clinical Pharmacology, Hannover Medical School, Hannover, Germany; 2 Central Hospital of the German Armed Forces (Bundeswehrzentralkrankenhaus), Rübenacher Straße 170, 56072 Koblenz, Germany

Remdesivir is an antiviral agent which is under clinical investigation for the treatment of coronavirus disease 2019 (COVID-19) [1]. Besides hypotension, deep-vein thrombosis, and delirium, rash has been reported as adverse drug reaction (ADR) of remdesivir [2]. The term “rash”, although commonly applied in clinical trials, is too unspecific to allow for a differentiated cutaneous ADR (cADR) management.

Symmetrical drug–related intertriginous and flexural exanthema (SDRIFE) is an immune-mediated cADR which occurs after systemic administration of a drug in an individual without prior sensitization [3, 4]. SDRIFE is clinically defined by five criteria: (i) onset after initial or repeated exposure to a systemically administered drug (contact allergens excluded), (ii) sharply demarcated erythema in the gluteal/perianal area and/or V-shaped erythema in the inguinal/perigenital region, (iii) involvement of at least one other intertriginous/flexural fold (e.g., axillae, antecubital fossae), (iv) symmetrical distribution, and (v) absence of systemic involvement [4]. Beta-lactam antibiotics, especially amoxicillin, are recognized as the most frequent trigger of SDRIFE [4, 5]. The clinical course of SDRIFE usually is benign and self-limited. SDRIFE may, however, progress to a generalized maculopapular exanthema if the eliciting drug is not withdrawn [6]. Treatment of SDRIFE consists of discontinuation of the offending agent. Additionally, antihistamines and topical/systemic glucocorticosteroids may provide symptomatic relief and speed up the healing process [5, 7, 8].

A 67-year-old male patient suffering from COVID-19 was transferred from Northern Italy to the Central Hospital of the German Armed Forces (Bundeswehrzentralkrankenhaus) Koblenz, Germany, on March 29, 2020, as part of an international medical evacuation operation. The patient had been mechanically ventilated since March 19 due to acute respiratory distress syndrome (ARDS). In Italy, the patient had been treated with intravenous remdesivir on a compassionate-use basis for four consecutive days (March 26 to 29). On March 30, the patient developed an erythematous macular exanthema on his trunk with pronounced involvement of both axillae, the inguinal and submental region. Remarkably, serous bullae with a diameter of up to 6 cm emerged in his axillae. The patient was physically examined by a senior dermatologist who established the diagnosis SDRIFE. Besides remdesivir, the patient was or had been treated with sufentanil, propofol, norepinephrine, pantoprazole, macrogol, metoclopramide, and enoxaparin. To evaluate the likelihood of each drug with regard to SDRIFE, we applied the Naranjo ADR Probability Scale (scale ranging from − 4 to + 13, with higher scores indicating a higher probability) [9]. The score for remdesivir was + 3 and for sufentanil, propofol, norepinephrine, pantoprazole, macrogol, metoclopramide, and enoxaparin was + 1, respectively, suggesting remdesivir as the primary offending agent. For a comprehensive medication chart of our patient as well as a detailed calculation of the score of each administered drug on the Naranjo ADR Probability Scale, please refer to Supplementary Table 1. Because of the concomitant viral ARDS, we refrained from glucocorticosteroid application. SDRIFE resolved spontaneously over the following weeks without further intervention.

PubMed was searched for case reports/series of SDRIFE in order to update preexisting lists of SDRIFE-eliciting drugs [3–5, 10, 11]. All identified publications were reviewed, and SDRIFE-eliciting drugs were extracted. We provide a comprehensive summary of currently known triggers of SDRIFE (Table 1).

**Table 1:** Reported causative agents of symmetrical drug-related intertriginous and flexural exanthema (SDRIFE)

Analgesics and nonsteroidal anti-inflammatory drugs [6, 10–15]	
Celecoxib, codeine, etoricoxib, ibuprofen, mefenamic acid, naproxen, nefopam, oxycodone, paracetamol, piritramide, salsalate	
Antiinfectives	
Antibiotics [4, 7, 8, 10, 11, 16–22]	
Amoxicillin, amoxicillin–clavulanate, ampicillin, ampicillin–sulbactam, benzylpenicillin, cefixime, ceftriaxone, cefuroxime, cephalexin, ciprofloxacin, clarithromycin, clindamycin, cloxacillin, trimethoprim–sulfamethoxazole, daptomycin (?), doxycycline, erythromycin, metronidazole, phenoxymethylpenicillin, pivampicillin, pristinamycin, roxithromycin, secnidazole	
Antifungals [5, 10, 23]	
Fluconazole, itraconazole, nystatin, terbinafine	
Antivirals [24, 25, our case]	
5-Fluorouracil (top.), remdesivir (?), valaciclovir	
Antisecretory drugs [3, 26, 27]	
Cimetidine, omeprazole, ranitidine	
Biologics [10, 28–30]	
Brentuximab vedotin, cetuximab, golimumab, infliximab	
Glucocorticosteroids [20, 31, 32]	
Betamethasone, cloprednol, deflazacort, dexamethasone, hydrocortisone, prednisolone (?), methylprednisolone (?)	
Oncologics [10, 33–35]	
Bortezomib, everolimus, gefitinib, mitomycin C	
Psychopharmaceuticals [10, 36–39]	
Clozapine, ethyl loflazepate, hydroxyzine, risperidone, rivastigmine, varenicline	
Radio contrast media [10, 40]	
Barium sulfate, iomeprol (Iomeron®), iopromide (Ultravist®)	
Miscellaneous [3, 10, 11, 41–46]	
Allopurinol, ambroxol, berberine, bufexamac (top.), *Coix lacryma-jobi* (ingredient of Ibane®) (?), etonorgestrel (released from Nuvaring®), heparin (i.v.), hydroxyurea, intravenous immunoglobulin, musk antihemorrhoids ointment, pseudoephedrine, tacrolimus, telmisartan–hydrochlorothiazide, terazosin, thiamine disulfide (?), zoledronic acid	

*(?)*, possible association with SDRIFE; *i.v.*, intravenous; *SDRIFE*, symmetrical drug-related intertriginous and flexural exanthema; *top.*, topical

Depending on the clinical context, the differential diagnosis of SDRIFE includes other cADRs such as acute generalized exanthematous pustulosis, drug reaction with eosinophilia and systemic symptoms, and (multifocal) fixed drug eruption, as well as nondrug-related dermatologic conditions, e.g., tinea cruris and inverse psoriasis [5, 7, 11]. A plethora of cutaneous manifestations have been described in connection with COVID-19 [47], including an SDRIFE-like exanthema [48]. However, the clear temporal relationship between the start of treatment with remdesivir and the development of the exanthema as well as the improvement of the exanthema upon cessation of remdesivir lead us to the conclusion that our patient experienced an SDRIFE elicited by remdesivir. Nevertheless, we cannot rule out that the comedication contributed to the development of SDRIFE in the presented case.

## Electronic supplementary material

ESM 1 (DOCX 23 kb).
