# Supplementary material for: Remdesivir-induced symmetrical drug–related intertriginous and flexural exanthema (SDRIFE)? A case report with review of the literature
Source: Eur J Clin Pharmacol. 2020 Aug 6;77(1):141–4. doi: 10.1007/s00228-020-02955-4 (PMC7406697; doi:10.1007/s00228-020-02955-4)
Supplement: Supplementary file 1 — (DOCX 23 kb). [file 228_2020_2955_MOESM1_ESM.docx]

| **Supplementary Table 1**: Medication chart of our patient including assessment of the administered drugs on the Naranjo Adverse Drug Reaction Probability Scale | | | | | | | | |
| --- | --- | --- | --- | --- | --- | --- | --- | --- |
|  | **Remdesivir** | **Sufentanil** | **Propofol** | **Norepinephrine** | **Pantoprazole** | **Macrogol** | **Metoclopramide** | **Enoxaparin** |
| **Medication details** | | | | | | | | |
| Start of treatment | March 26 | March 19 | March 19 | n.a. | n.a. | n.a. | n.a. | n.a. |
| End of treatment | March 29 | Administration continued until transfer of the patient to a rehabilitation clinic on April 23 | April 4 | April 21 | Administration continued until transfer of the patient to a rehabilitation clinic on April 23 | Administration continued until transfer of the patient to a rehabilitation clinic on April 23 | April 17 | Administration continued until transfer of the patient to a rehabilitation clinic on April 23 |
| Route of administration | i.v. | i.v. | i.v. | i.v. | i.v. | via feeding tube | i.v. | s.c. |
| Dosage | March 26: 200 mg/d  March 27–29: 100 mg/d | 50–100 µg/h | 60–240 mg/h | 50–300 µg/h | 40 mg/d | 13 g/d | 10 mg/d | 40 mg/d |
| **Naranjo Adverse Drug Reaction Probability Scale [9]** | | | | | | | | |
| Are there previous conclusive reports on this reaction?  (Yes: +1; No: 0; Do not know: 0) | 0 | 0 | 0 | 0 | 0 | 0 | 0 | 0 |
| Did the adverse event appear after the suspected drug was administered?  (Yes: +2; No: –1; Do not know: 0) | +2 | +2 | +2 | +2 | +2 | +2 | +2 | +2 |
| Did the adverse reaction improve when the drug was discontinued or a specific antagonist was administered?  (Yes: +1; No: 0; Do not know: 0) | +1 | 0 | 0 | 0 | 0 | 0 | 0 | 0 |
| Did the adverse reaction reappear when the drug was readministered?  (Yes: +2; No: –1; Do not know: 0) | 0 | –1 | –1 | –1 | –1 | –1 | –1 | –1 |
| Are there alternative causes (other than the drug) that could on their own have caused the reaction?  (Yes: –1; No: +2; Do not know: 0) | –1 | –1 | –1 | –1 | –1 | –1 | –1 | –1 |
| Did the reaction reappear when a placebo was given?  (Yes: –1; No: +1; Do not know: 0) | 0 | 0 | 0 | 0 | 0 | 0 | 0 | 0 |
| Was the drug detected in the blood (or other fluids) in concentrations known to be toxic?  (Yes: +1; No: 0; Do not know: 0) | 0 | 0 | 0 | 0 | 0 | 0 | 0 | 0 |
| Was the reaction more severe when the dose was increased, or less severe when the dose was decreased?  (Yes: +1; No: 0; Do not know: 0) | 0 | 0 | 0 | 0 | 0 | 0 | 0 | 0 |
| Did the patient have a similar reaction to the same or similar drugs in any previous exposure?  (Yes: +1; No: 0; Do not know: 0) | 0 | 0 | 0 | 0 | 0 | 0 | 0 | 0 |
| Was the adverse event confirmed by any objective evidence?  (Yes: +1; No: 0; Do not know: 0) | +1 | +1 | +1 | +1 | +1 | +1 | +1 | +1 |
| Total score | +3 | +1 | +1 | +1 | +1 | +1 | +1 | +1 |

The Naranjo Adverse Drug Reaction Probability Scale represents a tool to quantify the likelihood of a drug/multiple drugs to cause an adverse drug reaction in a given clinical context [9]. The scale ranges from –4 to +13, with higher scores indicating a higher probability. All dates refer to the year 2020. The patient developed symmetrical drug-related intertriginous and flexural exanthema (SDRIFE) on March 30.

I.v., intravenously; p.o., orally; s.c., subcutaneously; n.a., not available (indicating that the treatment was commenced before the transfer of the patient to our hospital); d, day; h, hour
